# Machine Learning Algorithms of Remote Sensing Data Processing for Mapping Changes in Land Cover Types over Central Apennines, Italy

**DOI:** 10.3390/jimaging11050153

**Published:** 2025-05-12

**Authors:** Polina Lemenkova

**Affiliations:** Department of Biological, Geological and Environmental Sciences, Alma Mater Studiorum—Università di Bologna, Via Irnerio 42, 40126 Bologna, Italy; polina.lemenkova2@unibo.it; Tel.: +39-3446928732

**Keywords:** time series, ecosystem, environmental monitoring, geoinformatics, satellite data, ecological conservation, multispectral image, Landsat, GRASS GIS, machine learning

## Abstract

This work presents the use of remote sensing data for land cover mapping with a case of Central Apennines, Italy. The data include 8 Landsat 8-9 Operational Land Imager/Thermal Infrared Sensor (OLI/TIRS) satellite images in six-year period (2018–2024). The operational workflow included satellite image processing which were classified into raster maps with automatically detected 10 classes of land cover types over the tested study. The approach was implemented by using a set of modules in Geographic Resources Analysis Support System (GRASS) Geographic Information System (GIS). To classify remote sensing (RS) data, two types of approaches were carried out. The first is unsupervised classification based on the MaxLike approach and clustering which extracted Digital Numbers (DN) of landscape feature based on the spectral reflectance of signals, and the second is supervised classification performed using several methods of Machine Learning (ML), technically realised in GRASS GIS scripting software. The latter included four ML algorithms embedded from the Python’s Scikit-Learn library. These classifiers have been implemented to detect subtle changes in land cover types as derived from the satellite images showing different vegetation conditions in spring and autumn periods in central Apennines, northern Italy.

## 1. Introduction

### 1.1. Background

Understanding and monitoring land cover dynamics in mountainous regions is critical for addressing the impacts of climate change, biodiversity loss, and socio-economic transitions [1,2]. These landscapes are shaped by complex interactions between natural processes and human activities, making them particularly sensitive to both environmental and anthropogenic drivers [3,4,5]. The Central Apennines of Italy, a biodiversity hotspot, is a prime example of such a region [6]. Its diverse ecosystems provide critical services, including water regulation, carbon sequestration, and habitat provision, while also supporting human livelihoods through forestry and agriculture [1]. However, these landscapes have undergone significant transformations over recent decades, driven by land abandonment, forest encroachment and climate-change related processes, including increased drought events and altered disturbance regimes [5,7,8].

Traditional agro-silvo-pastoral practices that once maintained a rich landscape mosaic have declined throughout the 20th century due to rural depopulation and socio-economic shifts [8]. Social factors include the aging of farmers and the relocation of young people to larger urban centres for better economic conditions [9,10]. This affected agricultural and agroforestry sectors in Apennines and caused changes in landscape mosaic, in a similar fashion to what observed in other mountainous landscapes [3,11,12,13]. The widespread agricultural land abandonment resulted in forest expansion and habitat homogenization. Over the 1936–2018 period, for instance, forest cover increased from 29% (1,673,266 ha) to 38% (2,214,671 ha) in Central Italy [14], and the area has been characterized as one of the land use change hotspots of Europe [15]. While some of these processes have positive effects, such as ecosystem restoration and increased forest cover, others pose significant challenges, including biodiversity loss, soil erosion, and reduced landscape diversity [16,17].

Effectively tracking these land cover dynamics is essential for informed conservation and management strategies [18,19]. However, traditional monitoring methods often rely on labor-intensive field surveys and fragmented data, which limit their spatial and temporal coverage [20,21,22]. Field-based studies, while valuable for localized and detailed insights, are inherently constrained by logistical challenges, including the difficulty of accessing remote or rugged terrains and the time-consuming nature of data collection [23]. Moreover, they lack the capacity to capture rapid or large-scale changes across diverse landscapes, making it difficult to establish comprehensive and timely assessments [24,25]. There is a growing need for advanced tools that can provide automated, scalable, and reproducible assessments of landscape changes.

Remote sensing (RS) technologies offer a transformative approach to landscape monitoring [26,27,28]. By providing spatially comprehensive and temporally consistent datasets, satellite imagery allows for detailed analysis of land cover patterns [29,30,31]. When coupled with Machine Learning (ML) algorithms, Remote Sensing (RS) data can be used to automate the classification and detection of land cover changes with high accuracy [32]. ML techniques are particularly advantageous in handling the complexity of RS data, enabling the identification of subtle patterns and trends that may be overlooked in traditional methods [33,34]. Despite these advantages, challenges remain in selecting the most effective ML algorithms for monitoring landscapes and ensuring the reproducibility of analyses.

The experience of the RS data applications to environmental monitoring of Apennines is recorded in many reported case studies [35,36,37]. These reflect a long process of technical evolution of Earth Observation (EO) data since 1970s and their applications for regional studies in Italy [38,39,40] and their practical application for ecological monitoring [41,42] and geological studies [43,44] of Italy. The case studies of EO-based mapping Apennines used for environmental monitoring went through a constant development of cartographic technologies and state-of-the-art methods of environmental monitoring [45,46,47,48]. The systematic, precise and operative observation of the landscape dynamics in Apennines using EO data began in late 20th century along with a progress in RS technologies, development of new software of their processing and general advances in mapping methods [49,50,51]. Since that period, environmentalists in Italy started recording changes in land cover types (deforestation, land fragmentation [52,53,54], soil erosion and systematical use of RS data processing [55]). Until now, GIS were employed for EO data processing and mapping in research focusing on Italian Peninsula [39,56].

Nevertheless, developing workflows to produce new data on land cover dynamics in a reproducible way is critical for understanding the long-term impacts of landscape transformations on biodiversity [57,58,59], ecosystem functioning [60], and local livelihoods [15,61]. Though existing studies Machine Learning (ML) has not been widely used in past studies of Apennines that use Remote Sensing (RS) data, our study revealed that ML combined with RS and thematic data, such as Coordination of Information on the Environment (CORINE), and several algorithms (such as RF, SVM, MLPC and DT), contributed to a more advanced research framework. Integrative approaches enable us to better understand landscape dynamics through revealed spatial-temporal trends in land cover types [62]. This is possible due to accurate computations, fine visualization, and precise image analysis supported by ML-based RS data analysis. Hence, integrative studies that employ several approaches and datasets play an important role in the environmental monitoring techniques. In this way, this paper contributed to development of advanced framework of geospatial data analysis with multi-source data and techniques.

Reproducibility and scalability are crucial for enabling comparative studies across regions and timeframes, facilitating the identification of broader patterns and trends. Scalability is particularly relevant in the context of accelerating environmental change, as it allows methodologies developed for the Apennines to be adapted and applied to other mountainous regions facing similar pressures. Developing automated workflows that leverage the strengths of ML algorithms and RS data allows the creation of the evidence base needed to inform conservation policies and land management practices tailored to the unique challenges of the Apennines, while also contributing to building a global framework for monitoring and managing landscape dynamics.

### 1.2. Objectives and Goals

Specifically, our objectives are:

1. To identify the most accurate ML algorithm for classifying and analyzing land cover changes in the region.
2. To apply this algorithm to quantify the main land cover changes from 2018 to 2022 in an automated and reproducible manner.

**Figure 1:**
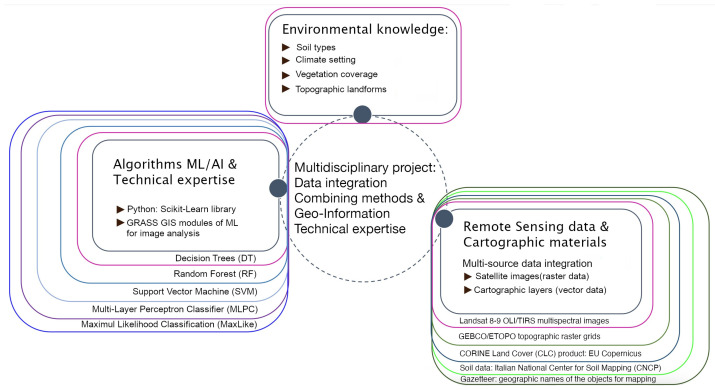
Conceptual approach of project workflow. Diagram source: author. Software: GIMP, version 3.0.2.

By achieving these objectives, this study seeks to advance the methodologies used in environmental monitoring and contribute to the conservation and management of mountainous landscapes using diversified conceptual approach integrating spatial data, technical methods and environmental knowledge, Figure 1.

In many cartographic cases, in biodiversity and land use change scenario evaluation are detected using time series analysis. However, these trends should be evaluated more using ML methods of automated image processing, because ML increases accuracy, speed and automation of data processing, which present significant advantages to geospatial analysis [63,64,65]. To address these issues, current work proposes the integration of ML methods, RS data analysis and environmental analysis for cartographic workflow. Specifically, the innovation introduced by the proposal consists in the ML-based classification of Landsat data. In this way, this study integrates three scientific domains: (1) computer science and ML algorithms, (2) image analysis and processing for pattern recognition; (3) environmental and Earth sciences: monitoring landscape dynamics in selected region of Italy. The procedure of image analysis was performed using advanced scripting Geographic Resources Analysis Support System Geographic Information System (GRASS GIS) software, version 8.4.1 tat combines a module-based classification methodology. Scripting techniques enabled to produce individual maps of land cover changes based on remote sensing (RS) data.

## 2. Study Area

**Figure 2:**
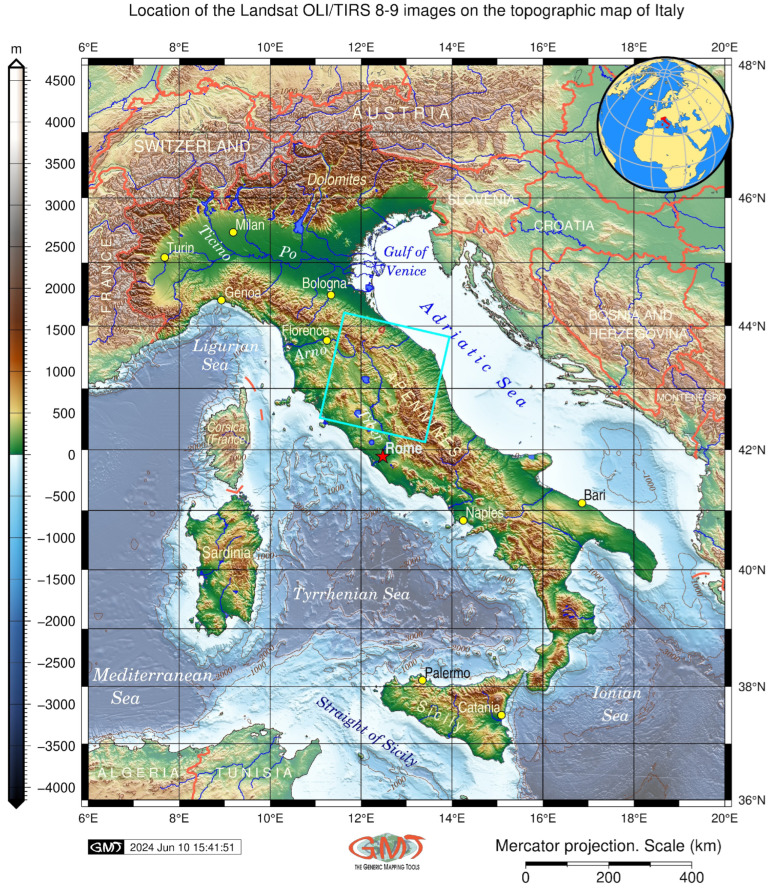
Topographic map showing the location of the study area. Blue square indicates the location of the Landsat satellite images within Italy; red star denotes the position of the capital. Map source: authors. Software: Generic Mapping Tools (GMT) version 6.5.0.

In this study, we aim to address these challenges by focusing on the application of RS and ML techniques to reconstruct land cover dynamics in the Central Apennines, Figure 2.

**Figure 3:**
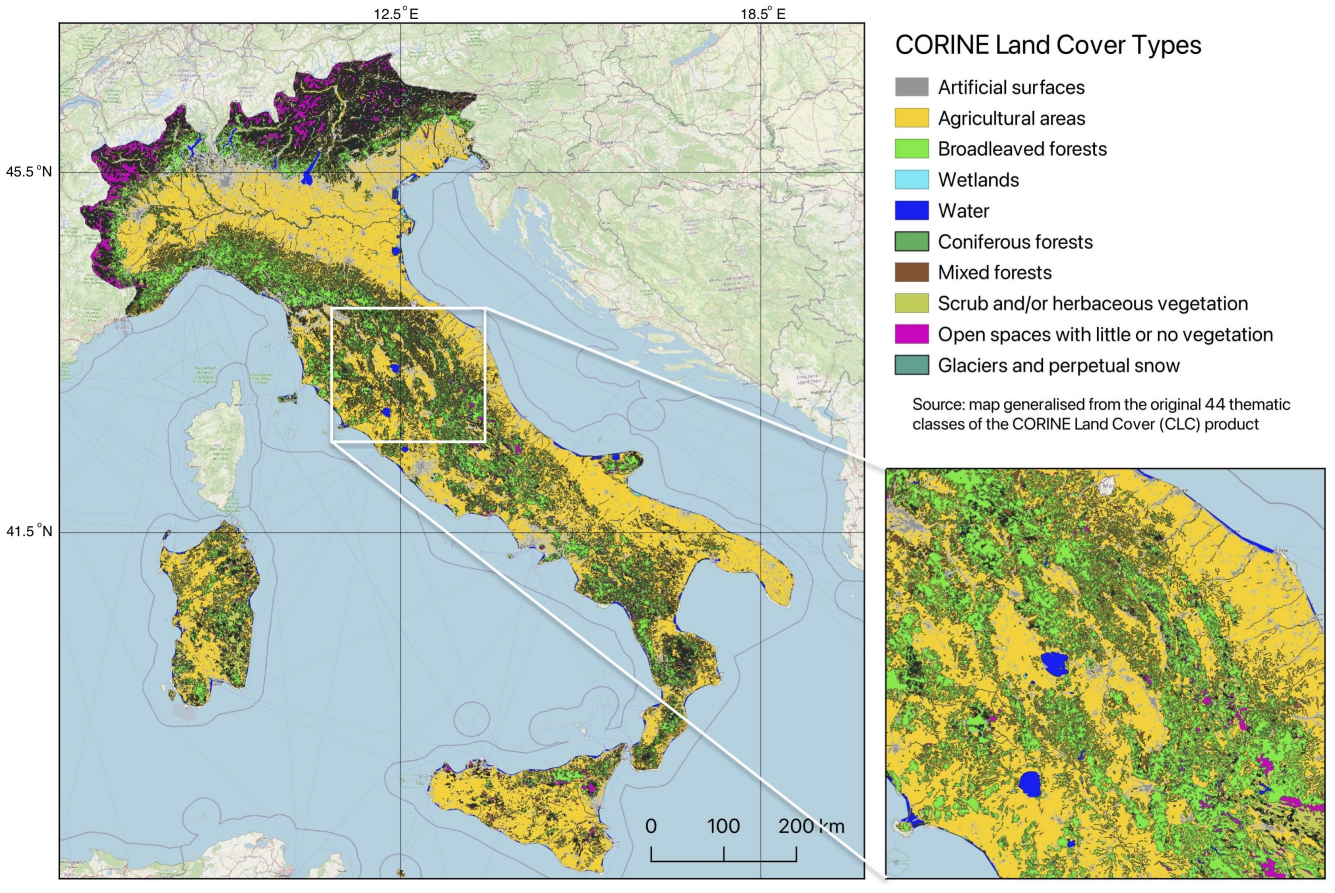
Land cover types in Italy: 10 major categories generalised from the original 44 classes of Coordination of Information on the Environment Land Cover Classification (CORINE CLC) with the increased fragment of the study area. Data source: Copernicus, 2018. Map source: author. Software: QGIS version 3.42.2 Münster.

Geographically, the study area covers the region of northern Italy and includes the provinces of Umbria, Marche, south-eastern part of Toscana and southern part of Emilia-Romagna, Figure 3. Factors that determine changes in biodiversity of Central Apennines are fundamental for land management in protected areas. Understanding reasons of environmental dynamics in context of land cover history and climate fluctuations plays a key role for proper land management policy and predictive ecosystem modelling in the Apennines. Climate fluctuations affect annual average temperature (ca. 8.9 °C), where the lowest values do dot exceed 8 °C at the top mountains, while the highest temperature (10 °C) is recorded in the lowlands. The average annual maximum is 11.5 °C, and minimum of 6.2 °C which consider as moderate for this region. The most common forest soils are cambisols with the bedrock consisting of sandstones and arenaceous marls (Geoportale Nazionale). Such types of soil are formed under the combination of topographic and climate setting including sufficient precipitation typical for the central Apennines, Figure 3. The information on the soil types has been collected from the data base with thematic maps showing soil regions of Italy, scaled 1:5,000,000 [66].

## 3. Methodology

This framework presents interdisciplinary study, since it strengthens the bridge between ecosystem studies and computer science through application of ML algorithms and image processing as effective tools for land cover analysis and biodiversity mapping. Specifically, advanced methods of RS data analysis that we apply in our study support monitoring goals to detect biodiversity changes or losses in the Apennines, and to evaluate the impacts this has land abandonment on ecosystem dynamics. In this way, RS data analysis enables to evaluate temporal changes in land cover, to map spatial distribution of patches within the landscapes that refer to the biodiversity, to identify regions which are most affected by changes and to quantify the percentage of these changes in six-year period (2018–2024) using tools of Geographic Resources Analysis Support System (GRASS) Geographic Information System (GIS) for pixel-wise image processing. In general aspect, our approach contributes to the substantially and conservation goals in northern Italy.

### 3.1. Data

The remote sensing data used in this study includes eight satellite images Landsat 8-9 OLI/TIRS collected from the United States Geological Survey (USGS) online repository. Remote sensing (RS) encompasses a wide range of datasets. Examples of RS data include Sentinel-2, ASTER, FORMOSAT-2, WorldView-2, VHR commercial images, Landsat 8-9 OLI/TIRS, and many other data from diverse sensors. Since the selection of data is the foundation of every research project, in this study we have chosen dataset that stands out for robustness, availability and applicability to research objectives and goals. The availability of Landsat data (coverage, cloudiness, appropriate data gathering for time series analysis) and its reputation explains the choice of these satellite images for environmental mapping. Besides, GRASS GIS software has necessary modules for processing Landsat formats. Therefore, in this study, we employed Landsat time series as reputable and robust source of information.

**Figure 4:**
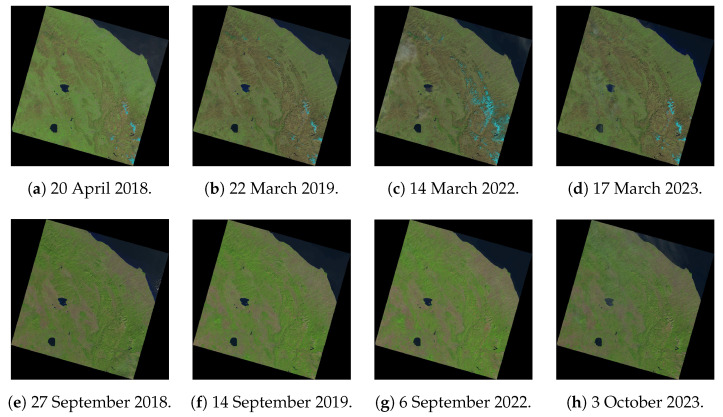
Original dataset of Landsat images in natural colour composite covering the region of Central Apennines. Data source: United States Geological Survey (USGS). Compilation: authors.

Landsat products have been utilised in diverse environmental applications during the past 40 years since the launch of the satellite mission. Its diverse sensors (MSS, TM, ETM+, and OLI) have been successfully reported in various studies [67,68,69,70]. Recently, Phiri and Morgenroth [71], for instance, evaluated how well different Landsat sensors performed in the tasks of land cover classification. Robust spatial resolution, availability of multispectral and panchromatic bands, high radiometric resolution, world coverage and the availability of quality data create reputation of the mission. Together, they are the main factors of active use of Landsat products in Earth and environmental studies [72,73,74,75,76,77,78]. Following the existing successful cases, in this study employs a time series of Landsat images. The overview of this dataset in natural colour composite is presented in Figure 4.

The essential characteristics and key feature attributes of the Landsat 8-9 OLI/TIRS images are summarised in the Table 1.

Technically, the data were obtained from the United States Geological Survey (USGS) which presents a collection of the diverse geospatial datasets in the open source repository. The row/path of the Landsat swath is 30/191 for all the scenes, collected in Nadir. The images are projected automatically in the UTM Zone 33 for Italy in WGS84 Ellipsoid/Datum and have an area with an extent of 185 × 185 km. When processing the Landsat scenes, only the multispectral bands were applied for image classification, since panchromatic bands are not relevant for research objectives of the current study. Major challenges facing a successful classification of the RS data are cloudiness of the satellite images, coverage, data availability and resolution which enable to accurately detect the changing features over the landscapes. Therefore, we selected the quality satellite data with low cloudiness and optimal coverage of the study region. Specifically, we used the moderate resolution multispectral satellite images obtained from the Landsat Collection Level-2.

The validation of data has been performed using the CORINE materials. The CORINE Land Cover inventory has a pan-European coverage and includes 44 thematic classes for the reference. Besides, it is widely accepted [79,80,81] that CORINE dataset is well-suited for regional vegetation monitoring studies, because it includes key environmental information on major land cover types. Such advantages of the CORINE explain their applications in environmental case studies covering diverse regions of Europe [82,83,84,85]. Nevertheless, the resolution of vector CORINE data is 100 × 100 m in their full coverage [86]. In view of this, the use of raster Landsat data with 30 × 30 m resolution upscales and details the existing land cover mapping. Besides, using new information retrieved from spaceborne images as time series provides new information derived from satellite images. Therefore, here we used the effective combination of vector (CORINE) and raster (Landsat) data for land monitoring of Apennines.

### 3.2. Workflow

In this workflow, we apply multiple ML classifiers (Random Forest, Support Vector Machine, Decision Tree, Multilayer Perceptron Classifier) due to their effectiveness and robust approach to RS data analysis using GRASS GIS version 8.4.1 (current stable release). Classifying RS data is a not straightforward task. Often the problems are caused by the methods of pattern recognition in raster scenes. For instance, high volume of data, requiring robust storage [87,88], effective transmission [89,90,91], and computational resources [92,93], to mention a few. The main challenge of satellite images classification is required high level of automation and large volume of data. The strengths of the ML algorithms when applied to RS data include their ability to process high-dimensional geospatial data, automate image analysis, and improve accuracy in complex tasks of data handling like classification and prediction. The limitations of the ML algorithms consist in the need for large amounts of high-quality training data, potential interpretability issues with complex models, and computational requirements. Nevertheless, the effectiveness of ML in RS data processing is reported in many existing case studies, e.g., see [94,95,96,97] Therefore, the rationale behind choosing the ML methods is justified by the suitability and effectiveness of ML for handling spaceborne images.

**Figure 5:**
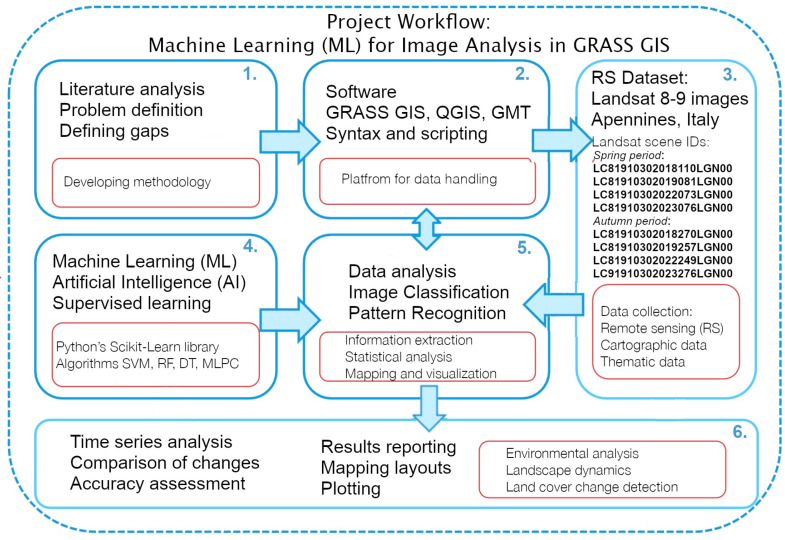
Workflow scheme of major research clusters and project management contexts. Diagram source: author. Software: GIMP, version 3.0.2.

The project workflow is illustrated schematically in Figure 5. The initial step included data import (collection from the USGS library) and preprocessing. Since details in the image may be obscured by technical noise, a pre-processing is required prior to image analysis, with aim at enhancing the quality of the data. Remote sensing data such as radar images include extended steps of pre-processing. For instance, speckle filtering is an approach of reducing granular noise in radar imagery. It has a salt-and-pepper pattern that frequently appears on the scenes due to the interference of waves reflected from a target object. For multispectral data, the GRASS GIS suite includes several modules, specifically adjusted to improving quality of Landsat data.

The data preprocessing was implemented using radiometric calibration, geometric correction and atmospheric correction. The latter was performed using the ‘i.landsat.toar’ modules that calculates top-of-atmosphere reflectance and transform the calibrated digital number (DNs) to top-of-atmosphere values. The geometric correction was performed using the ‘i.rectify’ module of GRASS GIS that calculates a transformation matrix for the improved scene. The improved data enabled to extract key features including the spectral reflectance and surface topology. The cloud masking has been embedded during the data collection as mask for lower than 10% of cloudiness.

**Figure 6:**
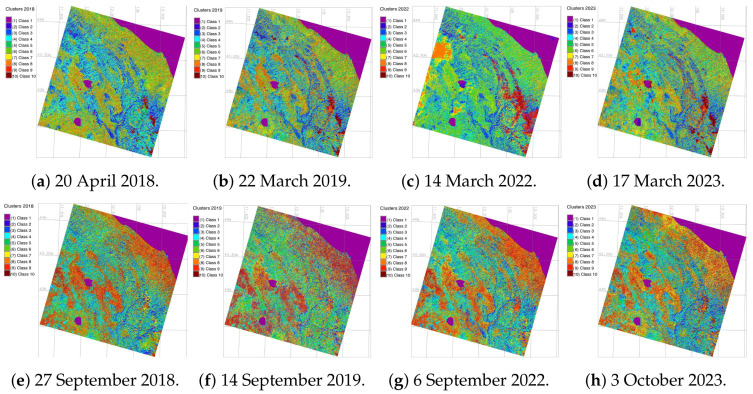
Images classified by the MaxLike algorithm for spring and autumn periods.

After data import and preprocessing which included radiometric calibration, geometric correction, speckle filtering and terrain correction, the images were first classified using the ‘MaxLike’ approach of the unsupervised classification which uses clustering, Figure 6. The unsupervised classification was done to receive the results from the MaxLike approach, to compare it with supervised classification. The training data seed was applied for ML processing of each scene and used for four approaches—Random Forest (RF), Support Vector Machine (SVM) Classifier, Decision Tree (DT) Classifier and Multilayer Perceptron Classifier (MLPC).

The classification of the pixels has been done using thresholding technique of remote sensing where threshold is a parameter used in image analysis. It is defined as the level of similarity which pixel has (digital number according to spectral reflectance) and according to this it is classified to the given class. Classifying each pixel on the raster matrix is based on its intensity level compared to the threshold value for the given class. The outcomes are validated with the computed reject threshold results using chi square test to determine levels of confidence of correctly classified pixels on raster scenes. Afterwards, the images were classified accordingly using the four ML algorithms for years 2018, 2019, 2022 and 2023). Full shell scripts of the GRASS GIS used for RS data processing (for each of 8 satellite images) and the obtained statistical results are placed in the GitHub repository: https://github.com/paulinelemenkova/Apennines (accessed on 7 April 2025).

### 3.3. Generalisation of CORINE Land Cover Map

The CORINE land cover map has been generalised from the initial 41 classes covering the whole country into 10 major classes, as necessary for the study area, Table 2. This has been done using the dissolve command in QGIS (QGIS 3.42.2 ‘Münster’), based on the attribute table. All the Class 1 category which is represented by the artificial classes was merged at Level 1 (category ‘1xx’ in CORINE classification). Following that, Class 2 ‘Agricultural areas’ was merged from the sub-classes such as 21x—arable lands, 22x—permanent crops, 23x—pastures and 24x—heterogeneous agricultural areas. The spatial resolution of CORINE is 25 ha/100 m.

Class 3 represents category ‘Forest and semi natural areas’. Here we performed the following generalisations in the classification. The sub-classes ‘311 Broadleaved forest and semi-natural areas’, ‘312 Coniferous forest’ and ‘313 Mixed forest’ were kept separated, since they represent important key classes that should be distinguished and recognised in our study area. In contrast, the sub-classes ‘32x—Scrub and/or herbaceous vegetation associations’ were merged. Specifically, these included 4 categories of CORINE—‘321. Natural grasslands’, ‘322. Moors and heathland’, ‘323. Sclerophyllous vegetation’ and ‘324. Transitional woodland-shrub’, since it is difficult to distinguish on the satellite images the slight differences in spectral reflectance between these categories. The Class ‘33x—Open spaces with little or no vegetation’ was merged of the sub-categories ‘331. Beaches, dunes, sands’, ‘332. Bare rocks’, ‘333. Sparsely vegetated areas’, and ‘334. Burnt areas’. The subclass ‘335. Glaciers and perpetual snow’ was kept separated since some satellite images contain snow-covered areas in the high mountains. Finally, wetlands (category ‘4xx’—all wetlands and their sub-categories) were merged together into one class. New classes were exported to shape files using ‘Export/Save Features as’ command in the QGIS.

### 3.4. Techniques and Algorithms

The satellite image processing was performed using Geographic Resources Analysis Support System (GRASS) GIS which provides the options of ML for RS data processing. The practical solutions of GRASS GIS to use the ML algorithms is derived from the Scikit-Learn Library of Python [98] and consists in using the embedded techniques where the name of each model is explicitly defined [99,100,101]. The ML methods of image processing include the following ones: (1) Random Forest (RF); (2) Support Vector Machine (SVM), (3) Decision Tree Classifier (DTC), and (4) Multi-layer Perceptron (MLP) Classifier of the Artificial Neural Network (ANN). These approaches enable to detect changes in land cover types through advanced image classification. These algorithms were tested to detect land cover changes and to select the most accurate classifier. We first run a maximum likelihood (MaxLike) classification. This method aims at the estimating the probability pixels distribution in the dataset, that is, cells within the satellite image and enables a straightforward approach to partition of pixels into groups using clusters of cells, Figure 6.

Technically, image classification was done using data on DNs of the pixels that vary in spectral reflectance according to different land cover types (e.g., water, forest, urban areas, meadows etc.). Similar spectral reflectance within the observed dataset are grouped into the same cluster and vice versa. The results of the MaxLike approach were employed as seed training dataset for the ML algorithms which are described in the following subsections.

### 3.5. Accuracy Assessment and Comparison of Four Algorithms

Since classification maps invariably contain misclassified pixels and possible classification mistakes, accuracy assessment is an essential step in any remote sensing-based classification effort. Besides, we compared the performance of four classification algorithms (RF, SVM, DT and the MLPC): Tables 4–7. Numerous factors cause possible errors, including the following ones:

- the type of input raw datasets used in image processing: Landsat OLI/TIRS data;
- the quality and details of the spectral signatures of training data (30-m resolution);
- the performance of the ML algorithms in processing on pixels in multispectral data;
- the complexity of the land cover pattern which affect the distinguishability of classes;
- the hierarchical level of the separability categories (10 classes), Appendix A.

The aim of accuracy assessment is to quantitatively evaluate the proportion of pixels correctly assigned to a given land cover category. Therefore, we evaluated the quality of the output maps made with the four ML algorithms and validated against the ground truth data obtained from CORINE:

1. Overall accuracy of the four maps generated using four different algorithms for two seasons (spring and autumn) for 2018;
2. User’s accuracy of individual 10 classes in each of the classification maps;
3. Kappa coefficient for identification of misclassified and confused classes mixed with other categories.

**Figure 7:**
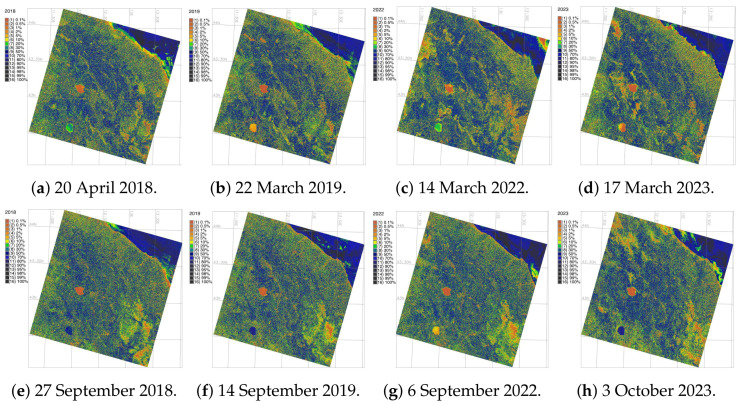
Accuracy assessment by rejection probability of pixels on the classified images using chi square test.

Cartographic accuracy of image classification is presented on the series of rejection probability maps. These maps visualise the reject raster maps which indicate the reject threshold results. The maps are generated based on the computed chi square test on each image analysis at various thresholds of confidence. This maps determines at what confidence level each cell is classified correctly. The visualised maps are presented in Figure 7 as the reject threshold layer which contains the index to one calculated confidence level for each classified cell in each Landsat image. The results of image classification were validated against CORINE Land Cover (CLC) data using GRASS GIS to evaluate the accuracy. The choice of CORINE is explained as follows. The CORINE CLC is a pan-European land cover database, offering a detailed classification scheme. The technical details, including metadata reference are available publicly on the Copernicus website: https://land.copernicus.eu/en (accessed on 5 May 2025).

The categories derived from CORINE serve as a ground truth data for validating results of Landsat classification. To perform validation, spatial overlay analysis was implemented using coordinates of the extracted fragment of the CORINE map against the classification maps. The area percentages of each land cover class in the Landsat classification was validated with the CLC data, following which the Kappa coefficient was calculated to assess the agreement between the image classification and the ground truth CLC dataset.

The user’s accuracy is computed as the number of correctly classified pixels in each land cover class related to total number of reference pixels in ground truth data in the same category. The overall accuracy indicates the total number of accurately classified pixels on the Landsat raster matrix related to the total number of reference pixel. Both overall and user’s accuracy are indicated in percentage (%) with the best values close to 100%. In contrast to these two approaches, Kappa coefficient is based on the chance-adjusted index of agreement where pixels are randomly assigned to classes and evaluated against the ground truth data. The assessment of accuracy after classification for validation of the results was performed using integration of thematic vector layers and RS datasets.

Clustering-based classification demonstrated the overall accuracy of the obtained maps for convergence of 98.1%, 98.2% and 98.1%, respectively. This demonstrated high mapping accuracy based on GRASS GIS processing of RS data. Among the accuracy methods, Kappa presents a discrete multivariate approach, Table 3. The evaluation of Kappa is normally suggested as moderate for values in the interval 0.41–0.60, acceptable for values from 0.61 to 0.80 and almost perfect agreement for values above 0.81. Spatial uncertainty in image classification shows the distribution of pixels to target classes which affects mapping results. It was estimated to avoid bias by the *chi* square test which plots the reject raster map. This map shows the level of threshold which determines confidence level at which each cell is categorised correctly and accurately assigned to the given class.

In the study area, 10 distinct land cover types were considered, as summarised in Table 2. Their CORINE codes, number of cells (pixels) in Landsat scenes and number of training samples were sued for ML validation, Figure 7. Nevertheless, the thematic aggregation of these 10 land cover classes cannot be directly linked to the parameters of vegetation and requires further studies. For RF algorithm, the number of correctly classified pixels (shown in bold black in Table 4) is 35,720. Divided by the total number of points 37,440, the overall classification accuracy for RF approach is 97%. The same approach was used for evaluation of classification correctness by other algorithms.

Table 4, Table 5, Table 6 and Table 7 show the error matrices that report the report the numerical data for the results of classification according to four tested algorithms.The tables show the relationship between the classified pixels in the satellite image and the corresponding ground truth data in the CORINE. According to the error matrices presented Table 4, Table 5, Table 6 and Table 7, the ML demonstrated robust accuracy for satellite image classification with high accuracy with the best performance is shown by the SVM algorithm slightly followed by the RF, DT and MLPC approaches.

For the SVM algorithm, the overall classification accuracy is 98% which shows the encouraging result with a higher accuracy. Other two algorithms, the DT and the MLPC also demonstrated reliable results, although lower values compared to the SVM and RF algorithms. Specifically, the overall classification accuracy for DT approach is 95% and for the MLPC algorithm, it is calculated as 96%. The demonstrated high classification accuracy levels were achieved due to the approach of ML as a key methodological approach that reduces the human-induced errors and possible misclassification. Specifically, accurate predictions on the test set using ML algorithms, indicates the correctly classifying model. Besides, robust quality of the initial dataset is also a factor that contributed to these results.

## 4. Results

The land cover types in the northern Italy were recognised as following 10 classes according to the existing adopted, modified and generalised classification schemes: (1) water areas, (2) arable lands and agricultural fields, (3) urban areas, (4) needle-leaved trees, (5) bare lands and soils; (6) pastures; (7) permanent herbaceous vegetation; (8) croplands; (9) broadleaved trees; (10) shrubland. The major differences verified between image analysis and cartographic data processing by ML methods regard agricultural lands, forests (broadleaved trees), croplands and shrubs, i.e., those coverings characterised by low human impact. For these types of land cover types, the levels of land abandonment and unsustainable pasture maintenance are high. Large areas, no longer used for farming activities, are prone to be spontaneously re-used by natural land cover types (i.e., secondary reforestation).

### 4.1. Comparison of the Four Algorithms

**Figure 8:**
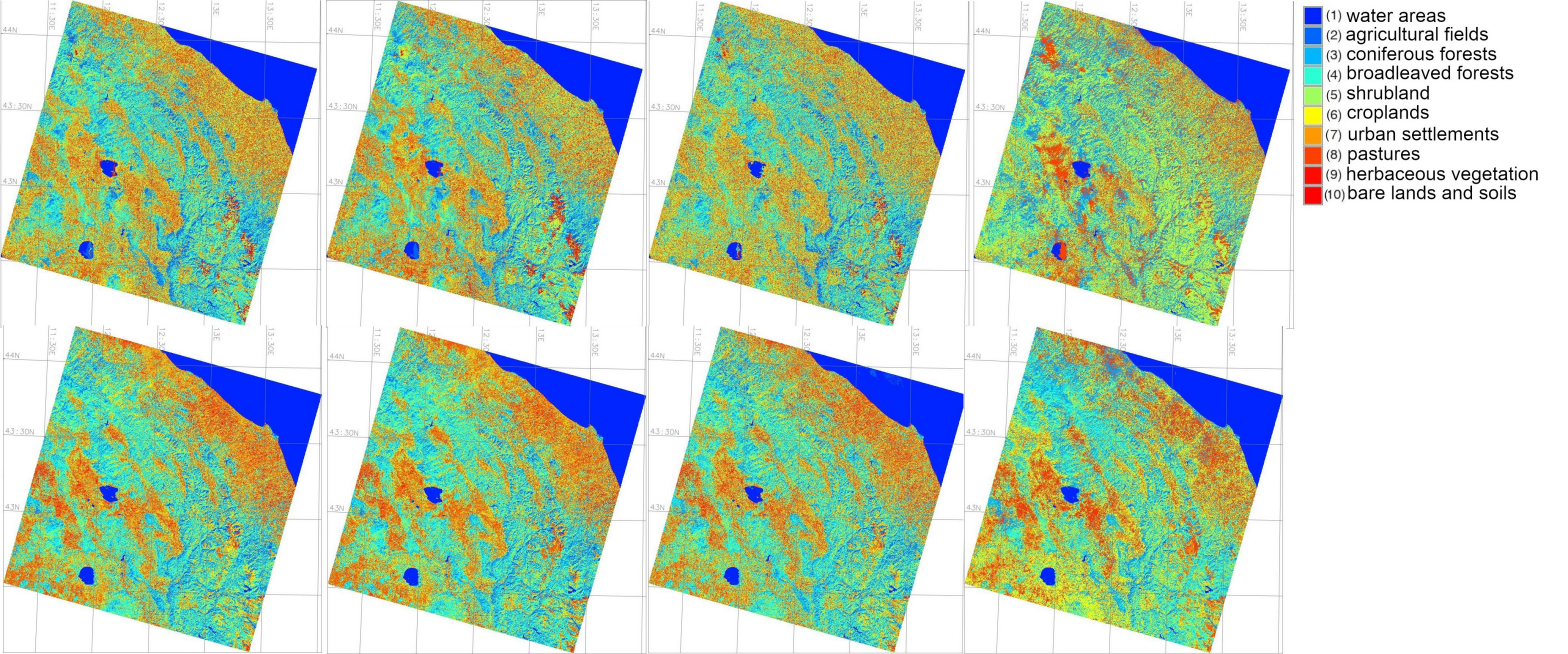
Land cover types in 2023 classified by four algorithms: Random Forest (RF), Support Vector Machine (SVM), Decision Tree (DT) and Multilayer Perceptron Classifiers (MLPClassifier) for spring and autumn periods.

The comparison of the four algorithms was performed using self-statistics based on the test data, as well as error matrices based on comparison with Corine Land cover data, Figure 8. The main land cover types identified included following 10 categories: (1) urban areas, (2) arable lands and agricultural fields, (3) water areas, (4) needle-leaved trees, (5) bare lands and soils; (6) pastures; (7) permanent herbaceous vegetation; (8) croplands; (9) broadleaved trees; (10) shrubland. The results show that the areas of arable lands and pastures are increasing. The exact areas covered by these land cover types are reported and can be explained by the increase of anthropogenic pressure.

More specifically, between 2018 and 2023, the total agricultural surface in the study area decreased by approximately 8.3715 ha (−16.65%), according to the data analysis from the ML methods of data processing. The areas occupied by the Class 2 (“arable lands and agricultural fields”) have decreased by 19.8 % from 3.77 to 2.64 ha which shows the minor decrease of cultivated and arable surfaces during this period. The extension of woodland is notable during the five-year period. In fact, according to the cartographic data, the difference between the areas occupied by forest and shrubland (Class 9 “broadleaved trees” and Class 10 “shrubland”) in 2018 and 2023 shows the slight increase of scrubland and shrubs, as well as broadleaved forests. Furthermore, woodland areas detected in the image analysis suggest that the woodland cover becomes greater.

**Figure 9:**
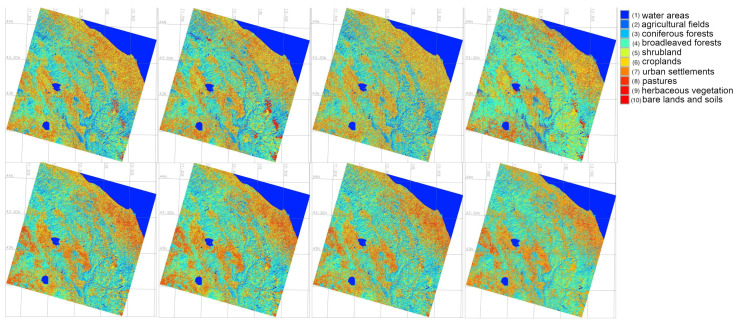
Satellite images on 2019 classified by four algorithms Random Forest (RF), Support Vector Machine (SVM), Decision Tree (DT) and Multilayer Perceptron Classifiers (MLPClassifier) for spring and autumn periods.

The areas covered by these land cover types were calculated and compared using four ML approaches to assess land cover dynamics. Using calculations of the landscape fragments, the regression and progression of forest areas were then assessed and reported in results of tabular form that show which type of the land cover types is expanding or decreasing. Comparative performance of algorithms is additionally evaluated using F-1 Score of Predictive Performance and Cohen’s Kappa Predictive Agreement were computed for ML models in GRASS GIS: (1) Random Forest (RF), (2) Decision Tree, (3) Multilayer Perceptron Classifiers (MLPClassifier),(4) Support Vector Machine (SVM), Table 3. The cartographic analysis shows that Class 8 represented by 8) croplands in the study area decreased by 36.7% while the Class 7 “permanent herbaceous vegetation” increased by 17.5%, Figure 9.

The results indicate that the use of this algorithm coupled to the 30 m resolution multispectral bands of Landsat had the best performance compared to other machine learning models, respectively. Seasonal variations of pixels by 10 classes on 2019, 2022 and 2023 are summarised in Table 8.

Based on the best performing algorithm, we analysed the main trends in land cover development in the study period. This included the identification of the major trends (increase-decrease of certain landscape patterns) in the area covered by the 10 land cover classes over the study period. The comparison of spatial occupation of vegetation and urban areas is ensured by the comparability of the maps with SVM as the best algorithm.

Specifically, the SVM outperforms maps in terms of visualization accuracy and is well aligned with results obtained from land cover change variations and urban growth around Central Apennines: Decrease in areas occupied by bare lands and soils (Class 5) and pastures (Class 6); the increase in areas occupied by shrubland (Class 10) and arable lands and agricultural fields (Class 2) as well as slight variations of other land use classes, related to climate and environmental impact factors. In addition, there are uncertainties in the automated pixel grouping and visualized in the precision maps of Figure 7. However, the use of directed classification eliminates these errors by more precisely assigning pixels to different classes. As noted earlier, the decision tree classifier provides a more realistic approach to land cover classifications needed to better estimate classes with assigned pixels.

### 4.2. Main Trends in Land Cover Types

To better understand land cover types and their occurrence on the pattern of landscapes, we analysed the detected patches that demonstrated dynamics with climate data and other land monitoring products. Comparing the obtained maps with Copernicus Land Monitoring Service (CLMS) portfolio, the satellite-derived maps show the influence of resolution of the initial raw dataset and methods used for their processing. Thus, areas of shrubland was estimated for the six-year period and compared with CLMS data. These products are derived from the Copernicus Land Monitoring Service which were measured at the pan-European level. Additional maps of soil covering Italy at regional scale were used for understanding the distribution of broadleaved and coniferous forests, in which we noted the influence of soil moisture on the variability of vegetation types.

The availability of water had the impact on the distribution of arable lands and pastures. Besides, the variability of land cover types changed under the impact of climate conditions. For instance, variations in such meteorological characteristics as relative humidity, temperature, radiation, wind speed and direction, and vapour pressure deficit caused changes in vegetation health and vegetation coverage) during the evaluated period (2018–2024), Table 8. Class 1 “urban areas”. represents a case with increased areas of coverage. Compared to the other patches in studied region, it covered a larger area in 2023 as previously in 2018. The difference is less remarkable, when Class 5 “bare lands and soils” is included for comparison as regions occupied by impervious structures such as roads or artificial surfaces. For urban and industrial areas, the areas are slightly increased by 22% in 2023 compared to the period of 2018, whereby the seasonal difference is not as important as for areas covered by vegetation, Table 8.

**Figure 10:**
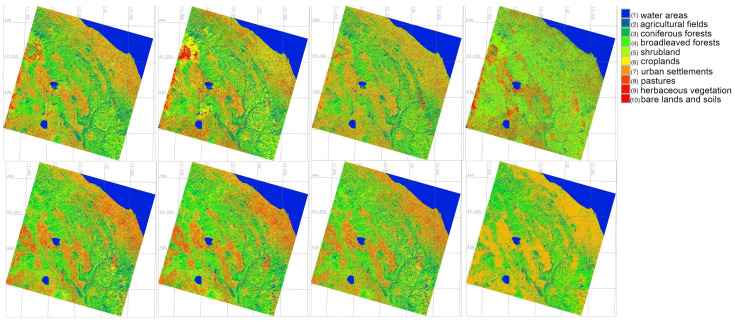
Satellite images on 2022 classified by four algorithms (Random Forest (RF), Support Vector Machine (SVM), Decision Tree (DT) and Multilayer Perceptron Classifiers (MLPClassifier)) for spring and autumn periods.

Technical differences in the algorithms of image processing are commented as follows. We compared the outputs of the classified images using algorithms of Support Vector Machine (SVM), Multi-layer Perceptron (MLP) Classifier of the Artificial Neural Network (ANN), Decision Tree Classifier (DTC), and Random Forest (RF), Figure 10. The uncertainties across the several ML techniques examined in this study (RF, SVN, DT, and MLPC) nearly always produced results that were acceptable, indicating that the analysis had a high degree of ML reliability, Figure 10. The best results are achieved by the SVM Classifier followed by the RF. Both algorithms differ from the DTC and ANN approaches, due to the different approaches in identification of the pixels’s value on the raster scenes (MLP of ANN and DTC). The maps generated using the SVM method are comparable with those based on the Random Forest Classifier, while MLP Classifier presented a more generalised map using deep learning network approach. The DTC demonstrated lesser quality of image processing compared to the above methods.

Compared to other approaches, the SVM algorithm demonstrated the best performance. It is general in nature and applies to classification of various types of land cover, including many types of vegetation. This algorithm is widely used to estimate certain environmental land-use properties and individual biophysical characteristics of the plant cover such as biomass and vegetation cover rate. The SVM approach aims to solve a complex classification by an optimal pixel transformation that determines the boundaries between cell points based on predefined classes. Its effectiveness is also presented in relevant works in which variables are retrieved from remote sensing data, including urban building detection, shadow detection or cloudiness values. The data processed by this method show a high level of reliability for the detection of land cover types. The SVM algorithm is used to drive process-based models and can be applied to support satellite image classification.

The advantage of SVM is explained by its effectivity in high dimensional datasets which is valuable for satellite images that consists thousands of pixels as cells. In our case, 8 multispectral images were classified automatically for each band of Landsat 8-9 OLI/TIRS scenes with 30-m resolution. The target periods cover spring and autumn periods with different vegetation types. Each raster image of the Landsat image contained 7911 rows and 7762 columns of cells, therefore, the automatic approach of processing there data was essential to ensure rapid and accurate data handling. Moreover, the SVM presents high search accuracy compared to other algorithms after a few iterations.

The SVM works well for time series analysis where several satellite images are compared, because this method is effective when number of dimensions is larger than samples. This is particular valuable for detecting complex land cover patterns in the regions of central Apennines with high heterogeneity of landscapes, such as mixed areas with urban areas and natural spaces. Being one of the most efficient inductive learning algorithms of ML, SVM has advantages in image classification due to the effects of dependence distribution of nodes within classes and integrated dependence of all nodes. In this way, it works similar to the Naïve Bayes, which makes it optimal for image classification where the dependences distribute evenly in classes. This excellently suits to the homogeneous types of landscapes with diversified land cover classes. However, this approach is not optimised for images with high landscape heterogeneity and contrasting landscape types (e.g., sharp gradient between the coastal and mountainous areas).

Following SVM, the RF classifier also presented optimal results of classification compared to the other approaches in the presented maps. Its superiority is explained by the tuned optimization of the image classification. In the case of RF, using the parameter tuning in GRASS GIS, each node is being split during the construction of a tree with the best split found either from all input pixels of the image or a randomised subset depending on the resolution and extent of the training dataset of the image which depends on the study area and heterogeneity of the visualised landscapes. This randomness aims at decreasing the variance of the RF estimator which allows to overcome overfitting of the individual decision trees with high variance in case of heterogeneous landscapes typical for Central Apennines. The artificially increased randomness of the RF algorithm enables either to decrease the prediction errors of the decision trees or cancel them out, thus making image classification more accurate. Furthermore, some observations regarding the various thematic classifications that were chosen can be made. For certain thematic aggregations of land cover classes, the differences between maps are less pronounced.

## 5. Discussion

Drivers of landscape change include diverse forces that can be divided into two categories: natural forces (climate and geological processes) and human activities (political, socio-economic and cultural transformation of landscapes, technological modifications of land use types). The integrated forces of these factors, taken together, shape landscapes and create unique mosaic patches, contribute to fragmentation and modified areas differently. Nevertheless, in various parts of the world, These factors affect landscapes with different force and intensity. For example, coastal areas are more prone to natural flood-related hazards, regions located in seismically active mountainous areas can be affected by geological hazards, e.g., earthquakes, landslides. Urban areas, such as rapidly developing cities and suburbans are largely influenced by social transformations of the society, such as urbanisation, re-structurisation of city spaces, etc. In this study, we demonstrated the regions that experience moderate impacts of these triggers in central areas of Italy that include mountainous regions of Apennines, coastal areas of the Adriatic Sea and populated areas around cities. Landscape transformation in such diversified regions as central Italy is dynamically developing process. Using RS data with advanced techniques supports monitoring such changes through controlling changes over time. We demonstrated the case of time series analysis of the satellite images to show the effectiveness of such approach.

The practical and operational implications of the presented work consists in application of novel methods of ML to image processing, thereby facilitating the real-time operative monitoring of Earth’s landscapes and increasing the availability of cartographic datasets for practical analysis of environmental changes in southern Europe. The role of ML is mainly to sustain the automation, precision and accuracy in the image analysis process, favouring rapid mapping in real-time regime, and increasing the availability of new environmental data. As we are now facing the era of big data, that is, large volume of geographical information with possible utilisation of these materials in the climate-environmental analysis, the possibility of generating series of maps and retrieval new information represents a critical element for ecosystem monitoring and landscapes regulation in the Apennine region. This evidence is in line with recent studies indicating the need for ML and automation in environmental analysis. In view of this, presented study supports environmental monitoring strategies and land management decisions in southern Europe.

The application of ML-based modelling to land cover change detection was made possible by the data integration, which was created for this study utilizing the methodology suggested in earlier ML-based case studies with satellite image processing for vegetation analysis. The GRASS GIS algorithm show accuracy for each change in land cover class, which can be quantified directly using numerical output. This algorithm is a type of ML that implies that the combination of the next best model and the previous model minimises the prediction error. Therefore, its effectiveness has been tested on satellite image processing with examples of Landsat and proved that it minimises the classification error through the defined target classification results. As the change results indicate that the forest and shrub areas in 2019 were partially lost or changed to other classes during the period up to 2023. On the contrary, the agricultural areas and shrubland have been developing in the past ten years.

Since the imagery data analysed only measures the land cover types of selected (restricted) territories covered by Landsat scene, they do not take into account social phenomena related to large cities and urbanised areas that were absent in the studied area. In turn, croplands and shrub lands are usually underestimated in land cover analysis because of the difficulties associated in detection small patches of shrubs on the satellite image with moderate resolution. Therefore, future studies might consider using diverse datasets to extent this analysis. For instance, other data might include Sentinel-2 images as RS data, or land cover datasets instead of CORINE, such as Google Dynamic World, ESRI Land Cover, and ESA WorldCover, ESA-CCI300, and Globeland30. The comparative analysis of different data and their applications can be considered as further directions of the undertaken study.Moreover, meadowlands can be in turn overestimated by the image analysis. On the other hand, diverse types of forests (among the broadleaved trees) are difficult to recognise using the Landsat scenes because of the similarities in spectral reflectance. Moreover, the changes resulted by the management of these lands and territories (e.g., conversion to other land use types) is challenging to detect by RS data. Therefore, the use of ML algorithms helped to overcome these difficulties through machine-based analysis.

## 6. Conclusions

The history of land cover/land use of the Central Apennines has been reported in previous studies. During recent decades, The landscapes of the Earth have been changing significantly due to the cumulative effects from climate change and human activities. Italy is one of the countries most vulnerable to such changes, especially in mountain areas. Pasture regions and woodlands have historically played significant role in the landscape mosaic of Central Apennines. However, from the middle of the 20th century, land cover structure, biodiversity and forest coverage in the Apennines has undergone notable dynamics. Two main drivers of ecosystem dynamics are climate change and anthropogenic processes. Anthropogenic factors affect landscapes through land exploitation and reshaped land cover patterns, as they are strongly related to social-economic development.

This research complements, in the environmental perspective, recent evidence that natural land cover types play a key role in environmental sustainability. Thus, settlement structure and patterns of agricultural fields replace natural vegetation that dominated in the landscapes in the past. Besides, social factors of changed land use pattern include decline in rural population that lead to land abandonment. Land abandonment lead to the expansion of forest area and habitat homogenization, decrease in landscape diversity, land use practices, and biodiversity. Such processes change of land cover structure in the Apennines since the middle of 20th century. Analysing landscape dynamics and the way how vegetated terrestrial ecosystems change over time is crucial for land management, policy makes and nature conservation.

Technically, this study attributed to development of advanced ML methods applied for RS data analysis as the linkage between the cartographic data processing and environmental monitoring in Central Apennines. Thus, we quantified the variability of land cover types in subalpine forests in the Central Italian Alps and assessed the landscape dynamics at basin level by combining different cartographic and ML image analysis approaches. The difference between land cover types was estimated for the target period (2018 to 2024) and presented as cartographic output based on time series analysis of the classified RS data. The uncertainty range of the image classification was evaluated using existing methods of statistical analysis including Kappa, F-Score and error matrices with calculated number of pixels that belong to each category in evaluated years. Technical methods to detect landscape dynamics remain unresolved topic in ecology and environmental mapping.

Biodiversity gradients have been analysed using diverse cartographic approaches along with the progress towards accurate explanatory modelling. As a contribution to the existing studies on environmental monitoring of Italy, this study presents the combination of ML techniques and RS data for ecosystem modelling. The computed differences in land cover change and maps prepared using ML-based image processing confirm the specialisation and the intensive exploitation of resources in the mountainous territories and hills rather than those in the lowlands in the study areas. These results also reflect the trend that has been taking place in various Italian regions since the middle of the 20th century and resulted in significant changes in the land cover pattern. Image processing is widely regarded as one of the most well studied areas in computer vision and environmental applications, yet recognising landscape patches under unconstrained effects from climate change affecting environmental landscapes in mountains of Central Apennines still remains an unsolved problem.

Remote sensing data can be successfully applied to identify different stages in vegetation and plant phenology as indicated by time series analysis. Specifically, detecting losses in mountainous ecosystems of Central Italy s possible using a sequence of satellite images taken for different years. Apart from the environmental aspect of the study, the applicability of RS data as satellite images as precious source of information at a regional scale is cartographically relevant. One cell on the Landsat image represents 30 × 30 m patch of landscape that differs from its surrounding environment. Unique features of landscape patches can be evaluated in terms of spectral reflectance of the given discrete area. Comparison of these patches depends on the size and the shape of satellite images that vary in resolution. In this regard, Sentinel-2 imagery can be further used to upscale geo-information. Thus, Sentinel-2 images will enable to evaluate distinct characteristics of heterogeneity of a landscape at more detailed level. Specifically for Italy, automatic landscape mapping and recognition of land cover types is a challenging task, because Apennine landscapes typically have large variations of patches, mosaic of vegetation inclusions intermittent with anthropogenic effects. Therefore, using satellite images with higher resolution would strengthen spatial analysis of the Apennines.

The paper demonstrated how the ML applications can be used for environmental monitoring and forest mapping, and how the choice of diverse ML instruments have driven their diverse outcomes of satellite image processing. The particular case of this work presented an example of the ML modules of GRASS GIS used to process the multispectral Landsat images for visualization of landscapes using image partition into 10 land cover classes automatically. The mapping example illustrates the region of central Apennines. For risk assessment and developing strategies in sustainable management of forests, the analysis of these territories requires further study. For instance, this can include thematic spatial models of change showing dynamic trends in agricultural lands and meadows. The processes of forestation and the abandonment of areas previously occupied by farmers can be developed in future studies based on the other datasets, e.g., Sentinel-2A imagery with higher resolution specifications.

Scripting provides a quantitative, robust and effective way of evaluating land cover properties through image processing and time series data analysis, which is a more advanced approach compared to traditional geographic fieldwork. To develop quick scripting codes in similar studies where GRASS GIS, R and Generic Mapping Tools (GMT) are applied in cartographic projects aimed at remote sensing data analysis, the full scripts are presented with provided comments and brief explanations of the commands. The reuse of these scripts may facilitate research in similar studies where processing of the satellite images and topographic data are required for environmental analysis. In this way, this study demonstrated the advantages of the scripting approach both for cartographic plotting and for satellite image processing, as well as plotting the flowchart diagrams for research design visualisation. Scripts of the GRASS GIS and GMT used as a standard methodological benchmark of automated image processing and cartographic workflow enabled to focus on the analysis of the landscapes of Apennines.

Mapping changes in land cover and assessing dynamics can show how a landscape pattern is distributed spatially. Therefore, in future research, it is advised to determine how effective the approach is at differentiating between the best thematic aggregation of maps and machine learning (ML) methods, as indicated by this study. Using automated data analysis, the mountainous regions of Central Apennines and surroundings were analysed through the classified series of images. The proposed algorithms of GRASS GIS scripts can be further extended to other regions and environmental applications where satellite image processing is required such as classification of landscapes using multi-temporal set of images. Hence, in this study, an advanced approach of GRASS GIS scripts was introduced to evaluating environmental properties of landscapes changed under climate and human-induced effects in Central Italy, using ‘i.cluster’, ‘i.maxlik’, ‘r.learn.train’, ‘r.learn.predict’ and other scripting modules of GRASS GIS. The output results shows the behaviour of land cover types in the coastal landscapes through the changes in patches as a result of the climate effects reacting as driver for land cover changes in mountainous ecosystems of Central Italy.

The consequences of various ML parameters on the cartographic outputs are analysed in this work, such as speed and accuracy, randomness of nodes, analytical determination of the output weights, and dependence distribution of pixels for each algorithm. Supervised learning models of GRASS GIS were tested and compared. The results shown that the most time-consuming algorithms was noted as SVC classification, while the fastest results were achieved by the Decision Tree approach to image processing and the best results are achieved by RF Classifier. Though each algorithms was developed to serve different objectives of ML applications in RS data processing, their technical implementation and practical purposes present valuable approaches to cartographic data processing and image analysis.

## Figures and Tables

**Table 1:** Technical parameters of the Landsat 8-9 OLI/TIRS images.

Landsat OLI/TIRS ^1^ Scene ID	Date Acquired	Cloud Cover % u.m.
Spring period		
LC81910302018110LGN00	20 April 2018	0.55
LC81910302019081LGN00	22 March 2019	0.67
LC81910302022073LGN00	14 March 2022	7.41
LC81910302023076LGN00	17 March 2023	2.52
Autumn period		
LC81910302018270LGN00	27 September 2018	0.26
LC81910302019257LGN00	14 September 2019	0.06
LC81910302022249LGN00	6 September 2022	0.06
LC91910302023276LGN00	3 October 2023	4.24

Information source: USGS. These data are provided from the Landsat sensors 8-9 Operational Land Imager (OLI) and Thermal Infrared Sensor (TIRS).

**Table 2:** Training ground truth samples of land types from CORINE.

Landsat OLI/TIRS Land Cover Class	CORINE Codes	Number of Pixels
Artificial surfaces	111–142	1,274,084
Agricultural areas	211–244	2,236,395
Broadleaved forests	311, 313–335	857,355
Coniferous forests	312	4,543,992
Mixed forests	313	47,634,298
Scrub and/or herbaceous vegetation	321–324	275,290
Open spaces with little of no vegetation	331–335	23,356
Glaciers and perpetual snow	335	14,891
Wetlands	411–423	23,714
Water	511–523	445,175

Information source: CORINE (Copernicus). The number of samples is smaller than the number of total pixels since a a representative training set is enough to train a ML classifier.

**Table 3:** F-1 Score of Predictive Performance and Cohen’s Kappa Predictive Agreement for ML models in GRASS GIS: (1) Random Forest (RF), (2) Decision Tree, (3) Multilayer Perceptron Classifiers (MLPClassifier), (4) Support Vector Machine (SVM).

**Spring**								
**Year**	**Cohen’s Kappa**				**F-1 Score**			
	**RF**	**DT**	**SVM**	**MLPC**	**RF**	**DT**	**SVM**	**MLPC**
2018	0.72	0.76	0.81	0.75	0.74	0.78	0.86	0.77
2019	0.79	0.78	0.86	0.72	0.81	0.86	0.90	0.81
2022	0.73	0.75	0.83	0.71	0.78	0.81	0.93	0.84
2023	0.79	0.78	0.82	0.74	0.75	0.85	0.83	0.79
**Autumn**								
**Year**	**Cohen’s Kappa**				**F-1 Score**			
	**RF**	**DT**	**SVM**	**MLPC**	**RF**	**DT**	**SVM**	**MLPC**
2018	0.78	0.72	0.81	0.76	0.74	0.71	0.90	0.78
2019	0.72	0.79	0.74	0.81	0.73	0.82	0.84	0.82
2022	0.85	0.81	0.76	0.82	0.79	0.82	0.84	0.83
2023	0.73	0.75	0.73	0.80	0.80	0.81	0.83	0.75

**Table 4:** Error matrix for validation of Support Vector Machine (SVM) algorithm.

Category	1: Urban Areas	2: Arable Lands	3: Water Areas	4: Needle-Leaved	5: Bare Lands	6: Pastures	7: Herbaceous	8: Croplands	9: Broadleaved	10: Shrubland	Row Total	Correct Pixels
Class 1	2589	48	62	1	0	50	2	18	0	2	2772	2589
Class 2	3	6359	27	0	5	1	1	2	0	0	6398	6359
Class 3	1	0	2908	1	32	47	0	0	10	5	3004	2908
Class 4	2	0	17	4818	9	7	14	0	2	0	4818	4869
Class 5	0	4	0	11	6138	4	3	0	0	0	6160	6138
Class 6	0	2	1	0	3	4281	12	21	0	0	4390	4281
Class 7	14	0	0	5	18	10	1407	9	28	22	1513	1407
Class 8	0	0	14	3	0	1	2	3389	0	9	3418	3389
Class 9	1	2	0	0	0	31	12	0	2409	0	2421	2409
Class 10	0	0	2	3	5	9	14	0	0	2513	2546	2513
Total	2600	6415	3031	4842	6200	4431	1517	3439	2439	2569	37,440	36,862

**Table 5:** Error matrix for validation of Random Forest (RF) algorithm.

Category	1: Urban Areas	2: Arable Lands	3: Water Areas	4: Needle-Leaved	5: Bare Lands	6: Pastures	7: Herbaceous	8: Croplands	9: Broadleaved	10: Shrubland	Row Total	Correct Pixels
Class 1	2175	73	51	0	42	72	12	27	6	7	2465	2175
Class 2	8	5886	25	14	31	18	0	0	1	4	5987	5886
Class 3	0	0	2924	5	27	35	0	12	0	9	3012	2924
Class 4	0	0	19	5102	4	0	2	15	13	0	5155	5102
Class 5	0	1	0	12	5957	4	11	25	1	0	6011	5957
Class 6	0	12	43	0	0	3984	24	0	0	1	4064	3984
Class 7	3	8	1	0	0	0	3106	12	17	32	3179	3106
Class 8	0	5	1	17	28	0	0	2396	0	31	2478	2396
Class 9	0	0	15	9	23	8	39	45	1965	10	2114	1965
Class 10	2	3	0	0	0	28	15	2	0	2925	2975	2925
Total	2144	6208	3247	5154	6117	4393	1474	3485	2523	2649	37,440	36,420

**Table 6:** Error matrix for validation of Decision Tree (DT) algorithm.

Category	1: Urban Areas	2: Arable Lands	3: Water Areas	4: Needle-Leaved	5: Bare Lands	6: Pastures	7: Herbaceous	8: Croplands	9: Broadleaved	10: Shrubland	Row Total	Correct Pixels
Class 1	2137	63	71	0	0	80	1	21	0	1	2373	2137
Class 2	5	6142	32	1	3	2	0	0	2	4	6191	6142
Class 3	0	0	3115	3	36	42	0	2	12	8	3218	3115
Class 4	0	0	23	5116	6	8	12	0	1	0	5166	5116
Class 5	0	2	0	15	6012	8	1	2	1	0	6041	6012
Class 6	0	0	4	0	58	3235	9	2	0	55	3363	3235
Class 7	1	0	2	0	36	8	1398	0	1	15	1461	1398
Class 8	0	1	0	2	3	4	0	3457	2	12	3481	3457
Class 9	1	0	0	12	6	3	32	0	2551	0	2605	2551
Class 10	0	0	0	5	7	3	21	1	0	2604	2641	2604
Total	2144	6208	3247	5154	6117	4393	1474	3485	2523	2649	37,440	35,720

**Table 7:** Error matrix for validation of Multi-layer Perceptron (MLP) Artificial Neural Network (ANN) algorithm.

Category	1: Urban Areas	2: Arable Lands	3: Water Areas	4: Needle-Leaved	5: Bare Lands	6: Pastures	7: Herbaceous	8: Croplands	9: Broadleaved	10: Shrubland	Row Total	Correct Pixels
Class 1	2095	68	63	2	0	15	0	0	1	12	2256	2095
Class 2	2	6371	38	0	0	21	14	0	1	0	6447	6371
Class 3	0	2	3487	2	18	27	0	0	0	12	3548	3487
Class 4	3	2	1	5279	2	9	24	0	0	0	5320	5279
Class 5	0	40	13	39	5364	0	0	22	0	11	5489	5364
Class 6	0	0	5	1	13	4106	1	14	21	0	4161	4106
Class 7	5	0	47	12	34	0	1405	0	31	62	1596	1405
Class 8	0	9	23	0	0	1	2	3188	0	4	3227	3188
Class 9	15	10	2	41	20	4	22	0	2356	0	2470	2356
Class 10	0	0	3	0	0	31	14	0	2	2376	2426	2376
Total	2144	6208	3247	5154	6117	4393	1474	3485	2523	2649	37,440	36,127

**Table 8:** Variations of pixels by classes on 2019, 2022 and 2023 in two different seasons.

**Spring**	**1: Urban Areas**	**2: Arable Lands**	**3: Water Areas**	**4: Needle-Leaved**	**5: Bare Lands**	**6: Pastures**	**7: Herbaceous**	**8: Croplands**	**9: Broadleaved**	**10: Shrubland**
2019	404	477	818	850	781	980	813	965	610	229
2022	398	433	981	775	732	892	900	963	521	240
2023	557	473	912	815	667	1128	955	900	622	291
**Autumn**	**1: Urban Areas**	**2: Arable Lands**	**3: Water Areas**	**4: Needle-Leaved**	**5: Bare Lands**	**6: Pastures**	**7: Herbaceous**	**8: Croplands**	**9: Broadleaved**	**10: Shrubland**
2019	680	342	725	645	854	682	622	629	858	313
2022	680	382	723	675	911	700	698	653	802	241
2023	720	341	696	673	1060	791	587	821	720	203

## Data Availability

The Landsat data used in this study are freely available from the USGS repository (https://earthexplorer.usgs.gov/ (accessed on 5 April 2025)). The GEBCO data used for plotting topographic map in Figure 1 is freely available in GEBCO repository (https://www.gebco.net/ (accessed on 3 April 2025)). The CORINE dataset is obtained from the Copernicus website (CORINE: https://land.copernicus.eu/en/products/corine-land-cover (accessed on 7 April 2025)). The codes used for data processing are available in the GitHub repository of the author: https://github.com/paulinelemenkova/Apennines (accessed on 8 April 2025).

## Abbreviations

ANN	Artificial Neural Network
CORINE CLC	Coordination of Information on the Environment Land Cover Classification
GIS	Geographic Information System
GMT	Generic Mapping Tools
GRASS GIS	Geographic Resources Analysis Support System GIS
Landsat 8-9 OLI/TIRS	Landsat Operational Land Imager/Thermal Infrared Sensor
ML	Machine Learning
MLPC	Multilayer Perceptron Classifier
RS	Remote Sensing
RF	Random Forest
SVM	Support Vector Machine
DTC	Decision Tree Classifier
USGS	United States Geological Survey

## Appendix A. Class Separability Matrices

(A1)
$$\left|\begin{array}{ccccccccccc} \; & 1 & 2 & 3 & 4 & 5 & 6 & 7 & 8 & 9 & 10\\ 1 & 0 & 0 & \; & & \; & & \; & & \; & \\ 2 & 2.9 & 0.0 & \; & & \; & & \; & & \; & \\ 3 & 3.9 & 0.7 & 0 & \; & & \; & & \; & & \\ 4 & 5.0 & 1.5 & 1.0 & 0 & \; & & \; & & \; & \\ 5 & 5.0 & 2.3 & 1.9 & 1.1 & 0 & \; & & \; & & \\ 6 & 5.1 & 1.4 & 0.7 & 1.0 & 1.9 & 0 & \; & & \; & \\ 7 & 5.2 & 2.0 & 1.4 & 0.8 & 0.9 & 1.1 & 0 & \; & & \\ 8 & 4.4 & 1.6 & 1.2 & 1.5 & 2.1 & 0.7 & 1.3 & 0 & \; & \\ 9 & 4.2 & 2.6 & 2.3 & 1.9 & 1.0 & 2.2 & 1.5 & 2.3 & 0 & \\ 10 & 4.0 & 3.5 & 3.3 & 3.2 & 2.8 & 3.3 & 2.9 & 3.2 & 2.1 & 0 \end{array}\right|\;\left|\begin{array}{ccccccccccc} \; & 1 & 2 & 3 & 4 & 5 & 6 & 7 & 8 & 9 & 10\\ 1 & 0 & \; & & \; & & \; & & \; & & \\ 2 & 2.4 & 0 & \; & & \; & & \; & & \; & \\ 3 & 3.5 & 0.9 & 0 & \; & & \; & & \; & & \\ 4 & 4.6 & 1.5 & 0.7 & 0 & \; & & \; & & \; & \\ 5 & 4.8 & 2.0 & 1.3 & 0.7 & 0 & \; & & \; & & \\ 6 & 4.5 & 2.5 & 1.6 & 1.4 & 1.3 & 0 & \; & & \; & \\ 7 & 4.3 & 2.4 & 1.7 & 1.4 & 1.0 & 0.6 & 0 & \; & & \\ 8 & 4.2 & 2.8 & 2.1 & 2.1 & 2.2 & 1.0 & 1.3 & 0 & \; & \\ 9 & 5.1 & 3.5 & 2.7 & 2.7 & 2.5 & 1.4 & 1.3 & 0.8 & 0 & \\ 10 & 4.4 & 3.4 & 2.9 & 2.8 & 2.7 & 2.0 & 1.8 & 1.5 & 0.9 & 0 \end{array}\right|$$

Class separability matrices between 10 classes: spring and autumn 2019.

(A2)
$$\left|\begin{array}{ccccccccccc} \; & 1 & 2 & 3 & 4 & 5 & 6 & 7 & 8 & 9 & 10\\ 1 & 0 & \; & & \; & & \; & & \; & & \\ 2 & 1.1 & 0 & \; & & \; & & \; & & \; & \\ 3 & 2.4 & 1.3 & 0 & \; & & \; & & \; & & \\ 4 & 3.4 & 2.3 & 0.9 & 0 & \; & & \; & & \; & \\ 5 & 4.3 & 3.1 & 1.7 & 0.8 & 0 & \; & & \; & & \\ 6 & 5.0 & 3.9 & 2.4 & 1.5 & 1.0 & 0 & \; & & \; & \\ 7 & 4.9 & 3.8 & 2.5 & 1.7 & 0.9 & 0.9 & 0 & \; & & \\ 8 & 5.8 & 4.7 & 3.3 & 2.5 & 1.8 & 1.0 & 1.1 & 0 & \; & \\ 9 & 5.5 & 4.6 & 3.4 & 2.8 & 2.3 & 1.7 & 1.7 & 0.9 & 0 & \\ 10 & 6.1 & 5.1 & 3.9 & 3.2 & 2.9 & 2.0 & 2.5 & 1.5 & 1.3 & 0 \end{array}\right|\;\left|\begin{array}{ccccccccccc} \; & 1 & 2 & 3 & 4 & 5 & 6 & 7 & 8 & 9 & 10\\ 1 & 0 & \; & & \; & & \; & & \; & & \\ 2 & 1.1 & 0 & \; & & \; & & \; & & \; & \\ 3 & 2.0 & 1.1 & 0 & \; & & \; & & \; & & \\ 4 & 2.9 & 2.3 & 1.2 & 0 & \; & & \; & & \; & \\ 5 & 4.1 & 3.7 & 2.6 & 1.2 & 0 & \; & & \; & & \\ 6 & 5.1 & 4.9 & 3.7 & 2.4 & 1.2 & 0 & \; & & \; & \\ 7 & 5.6 & 5.6 & 4.5 & 3.1 & 2.1 & 1.0 & 0 & \; & & \\ 8 & 6.2 & 6.4 & 5.3 & 4.1 & 3.1 & 2.1 & 1.1 & 0 & \; & \\ 9 & 7.8 & 8.5 & 7.3 & 5.9 & 5.0 & 3.9 & 2.8 & 1.6 & 0 & \\ 10 & 7.9 & 8.4 & 7.4 & 6.2 & 5.3 & 4.4 & 3.4 & 2.3 & 1.1 & 0 \end{array}\right|$$

Class separability matrices between 10 classes: spring and autumn 2022. [H]

(A3) $$\left|\begin{array}{ccccccccccc} \; & 1 & 2 & 3 & 4 & 5 & 6 & 7 & 8 & 9 & 10\\ 1 & 0 & \; & & \; & & \; & & \; & & \\ 2 & 1.2 & 0 & \; & & \; & & \; & & \; & \\ 3 & 2.0 & 1.2 & 0 & \; & & \; & & \; & & \\ 4 & 2.4 & 1.8 & 0.8 & 0 & \; & & \; & & \; & \\ 5 & 2.4 & 2.3 & 1.2 & 1.0 & 0 & \; & & \; & & \\ 6 & 2.7 & 2.5 & 1.6 & 0.9 & 0.9 & 0 & \; & & \; & \\ 7 & 2.8 & 3.1 & 2.3 & 1.9 & 1.1 & 1.2 & 0 & \; & & \\ 8 & 3.0 & 3.0 & 2.4 & 1.7 & 1.4 & 0.9 & 1.0 & 0 & \; & \\ 9 & 3.2 & 3.5 & 2.9 & 2.5 & 1.9 & 1.7 & 1.1 & 1.0 & 0 & \\ 10 & 3.1 & 3.8 & 3.1 & 2.9 & 2.1 & 2.2 & 1.2 & 1.8 & 1.1 & 0 \end{array}\right|\;\left|\begin{array}{ccccccccccc} \; & 1 & 2 & 3 & 4 & 5 & 6 & 7 & 8 & 9 & 10\\ 1 & 0 & \; & & \; & & \; & & \; & & \\ 2 & 2.6 & 0 & \; & & \; & & \; & & \; & \\ 3 & 4.0 & 0.9 & 0 & \; & & \; & & \; & & \\ 4 & 4.5 & 1.7 & 0.9 & 0 & \; & & \; & & \; & \\ 5 & 4.4 & 1.3 & 0.7 & 1.1 & 0 & \; & & \; & & \\ 6 & 4.0 & 1.5 & 1.2 & 1.7 & 0.8 & 0 & \; & & \; & \\ 7 & 5.0 & 2.1 & 1.4 & 1.0 & 1.0 & 1.2 & 0 & \; & & \\ 8 & 5.5 & 2.2 & 1.4 & 0.6 & 1.3 & 1.9 & 0.7 & 0 & \; & \\ 9 & 5.7 & 3.0 & 2.2 & 1.4 & 2.2 & 2.6 & 1.2 & 0.9 & 0 & \\ 10 & 5.2 & 3.3 & 2.7 & 2.2 & 2.6 & 2.9 & 1.9 & 1.7 & 1.1 & 0 \end{array}\right|$$

Class separability matrices between 10 classes: spring and autumn 2023.
